# Digital peer-to-peer support programme for informal caregivers of people living with motor neuron disease: study protocol for a multi-centre parallel group, single-blinded (outcome assessor) randomised controlled superiority trial

**DOI:** 10.1186/s13063-023-07124-3

**Published:** 2023-02-20

**Authors:** Louise Rose, Thilipan Thaventhiran, Esther Hobson, Rebecca Rogers, Kirsty James, Petrina Chu, Ben Carter, Christina Faull, Sian Saha, Jeong Su Lee, Georgios Kaltsakas, Christopher McDermott, Michelle Ramsay

**Affiliations:** 1 Florence Nightingale Faculty of Nursing, Midwifery and Palliative Care, King’s College London, James Clerk Maxwell Building, London, SE1 8WA United Kingdom; 2 Lane Fox Respiratory Service, Guy’s and St Thomas’ NHS Foundation Trust, Westminster Bridge Rd, London, SE17HE United Kingdom; 3 Sheffield Institute for Translational Neuroscience, University of Sheffield, 385a Glossop Rd, Sheffield, S102HQ United Kingdom; 4 Department of Biostatistics and Health Informatics, Institute of Psychiatry, King’s College London, Psychology & NeuroscienceDe Crespigny Park, London, SE5 8AF United Kingdom; 5 LOROS Hospice, Groby Rd, Leicester, LE39QE United Kingdom; 6 King’s College Hospital, Denmark Hill, London, SE59RS United Kingdom; 7 Centre for Human and Applied Physiological Sciences (CHAPS), King’s College London, London, SE1 8WA United Kingdom

**Keywords:** Peer support, Motor neuron disease, Amyotrophic lateral sclerosis, Informal caregiver, e-Health, Randomised controlled trial

## Abstract

**Background:**

Peer support is effective in improving psychological well-being of family caregivers of people with conditions such as dementia, cancer, and brain injury. However, there are limited data on effective psychological interventions for family caregivers of people living with motor neurone disease. Our objective is to evaluate the efficacy of a virtual peer support programme for improving caregiver psychological wellbeing and caregiving related outcomes.

**Methods:**

We will conduct a multi-centre parallel group randomised controlled superiority trial. Using a multi-modal recruitment strategy, we will recruit informal caregivers from UK MND clinics, in-patient units, and hospices. We will randomise (1:1, stratified by gender) participants to either a 12-week virtual peer support programme or usual care comprising provision of online information resources publicly available via the MND Association website. Peer support programme elements will be delivered via a secure digital e-platform aTouchAway™ (Aetonix, Canada). Our target sample size is 160 (80 each arm). Our primary outcome is the Hospital Anxiety and Depression Scale (HADS) assessed at 12 weeks (primary endpoint). Secondary outcomes that will also be assessed at 12 weeks include the Zarit Burden Interview, Pearlin Mastery Scale, Personal Gain Scale, Positive Affect Scale, and the Brief COPE. Outcome assessors will be blinded to allocation. Tertiary outcomes include perceived usability (1 item 9-point Likert scale) and acceptability (semi-structured qualitative interviews) of the peer support programme. Intervention fidelity measures will comprise frequency, type (text, audio, video), and duration (audio and video) of peer support contact downloaded from the aTouchAway AWS server. We will use a mixed-effects linear model to test the effect of the intervention on the primary outcome. Secondary outcomes will be analysed using linear regression. We have ethical approval (21/NW/0269) from the North-West Research Ethics Committee, UK.

**Discussion:**

This single-blinded randomised controlled trial will determine the effect of a virtual peer support programme on caregiver psychological wellbeing and caregiver burden. This study will examine the impact of a virtual peer support intervention on quality-of-life measures in informal caregivers of individuals with MND living in the community.

**Trial registration:**

ClinicalTrials.gov: NCT04695210

## Administrative information

Note: the numbers in curly brackets in this protocol refer to SPIRIT checklist item numbers. The order of the items has been modified to group similar items (see http://www.equator-network.org/reporting-guidelines/spirit-2013-statement-defining-standard-protocol-items-for-clinical-trials/).

**Table:** 

Title {1}	Digital peer-to-peer support programme for informal caregivers of people living with motor neuron disease: study protocol for a multi-centre parallel group, single-blinded (outcome assessor) randomised controlled superiority trial Protocol Short title: RCT of virtual peer-to-peer support programme for carers of people living with MND
Trial registration {2a and 2b}.	ClinicalTrials.gov: NCT04695210.
Protocol version {3}	Version 1.2; March 11, 2022
Funding {4}	Marie Curie Charity.
Author details {5a}	^1^ Florence Nightingale Faculty of Nursing, Midwifery and Palliative Care, James Clerk Maxwell Building, King's College London, London SE1 8WA, United Kingdom ^2^ Lane Fox Respiratory Service, Guy’s and St Thomas’ NHS Foundation Trust, Westminster Bridge Rd, London, SE17HE, United Kingdom ^3^ Sheffield Institute for Translational Neuroscience, University of Sheffield, 385a Glossop Rd, Sheffield, S102HQ, United Kingdom ^4^ Department of Biostatistics and Health Informatics, Institute of Psychiatry, Psychology & Neuroscience, King's College London, De Crespigny Park, London SE5 8AF, United Kingdom ^5^LOROS Hospice, Groby Rd, Leicester, LE39QE, United Kingdom ^6^ King’s College Hospital, Denmark Hill, London, SE59RS, United Kingdom ^7^ Centre for Human and Applied Physiological Sciences (CHAPS), King's College London, London SE1 8WA, United Kingdom
Name and contact information for the trial sponsor {5b}	King’s College London Name of Sponsor Representative: Reza Razavi Address: Rm 5.31 James Clerk Maxwell Building, 57 Waterloo Rd, London SE1 8WA, United Kingdom Telephone: 02,078,483,224 Email: reza.razavi@kcl.ac.uk Guy’s & St Thomas’ Foundation NHS Trust Name of Sponsor Representative: Rachel Fay Address: 16th Floor, Tower Wing, Great Maze Pond Telephone: 02,071,887,188 Email: R&D@gstt.nhs.uk
Role of sponsor {5c}	Role of sponsor includes local Research and Development approval, institutional indemnity insurance for the trial, but does not include funding or trial conduct

## Introduction

### Background and rationale {6a}

Across the world, patient care is frequently reliant on informal caregivers to provide care at home. An informal carer is someone who provides unpaid help and support to a dependent person with whom they have a social relationship. The type of informal care delivered is diverse, varying in terms of most responsible diagnosis, multimorbidity, complexity of care, cumulative time spent caring, and the availability of support/information [1]. In the UK, an estimated 2.1 million people take on a caring role for a family member or friend every year [2].

Motor neuron disease (MND) is a life-limiting neurological disease that has a terminal diagnosis and is characterised by progressive muscle wasting, gradual paralysis, and respiratory failure. Death generally occurs 2 to 5 years from diagnosis [3]. Rapid and unpredictable physical deterioration demands in addition to coping with distress require ongoing adaption of informal caregivers to assume increasing care responsibilities [4]. Although assistive technology such as mechanical ventilation supports independent living in the community reducing the need for formal health and support services, informal caregivers can experience an exceptional care burden and are less likely to be able to access existing personal support networks [5]. This means psychosocial support needs to be sought elsewhere.

Appropriate psychosocial support is a key enabler for caregivers to maintain their wellbeing and to continue providing care at home [6]. Psychosocial interventions include different modalities of psychotherapy and counselling, case management, and structured peer support. Two randomised controlled trials (RCT) conducted in the Netherlands investigated case management [7] or cognitive behavioural therapy [8] as interventions for family caregivers of people living with MND. Both found minimal impact of these interventions on outcomes such as perceived burden and quality of life. Caregivers of technology dependent people with MND may experience difficulties in accessing interventions that require in-person attendance because of geographic limitations, time constraints associated with caring, economic burden, and unavailability of respite [9, 10]. This in turn increases psychological distress.

Peer-to-peer support is based on the premise that individuals who have lived experience are in a unique position to understand and provide invaluable insight to individuals going through a similar experience in the present. Peer support consists of emotional (e.g. expressions of caring, empathy and reassurance), informational (e.g. advice, suggestions, factual input, and feedback), and affirmational (e.g. affirmation of feelings and behaviours, reassurance that frustrations can be managed) support [11]. The experiential and relational aspect of homophily-driven peer affiliation creates supportive relationships with potential to improve quality of life and wellbeing by decreasing feelings of isolation, improving mood, buffering stress, creating a sense of empowerment, and increasing self-efficacy. Peer support can also serve to inhibit maladaptive behaviours and promote adaptive coping strategies [12–15].

Web-based applications to support informal caregivers have emerged as cost-effective, accessible, and convenient interventions. Informal caregivers perceive online peer-support as a beneficial resource [16]. MND is a rare condition and carers may experience loneliness and isolation [17]. Support from people in similar circumstances can be encouraging. Therefore digital peer-to-peer support is a promising interactive psychosocial intervention that is accessible and convenient for people with high caregiving demands.

### Objectives {7}

The primary trial objective is to determine the effectiveness of a virtual peer-to-peer support programme on psychological distress of informal caregivers of people with MND as measured using the Hospital Anxiety and Depression Scale (HADS) which is collected at baseline, week 6 (mid-trial), and week 12 (end of trial participation). Secondary objectives are to determine the effect of the peer support intervention on caregiving outcomes including the effect on caregiver burden, mastery, personal gain, and coping strategies. Tertiary objectives are to determine process outcomes including intervention acceptability, usability, and fidelity.

### Trial design {8}

This is a non-commercial multi-centre, parallel group, single-blinded (outcome assessor) 1:1 randomised controlled superiority trial that aims to determine the efficacy of a 12-week virtual peer-to-peer support programme on informal caregiver psychological wellbeing and caregiver burden. We will include process measures to describe fidelity and qualitative semi-structured interviews to explore intervention acceptability.

## Methods: participants, interventions, and outcomes

### Study setting {9}

The virtual peer support programme will be delivered online and accessible to participants from their personal digital devices in the home. Recruitment sources are multi-modal and include but will not be limited to the following: MND clinics, home ventilation clinics, and hospices across England, Scotland, and Wales as well as via the UK MND Association, social media, and snowballing referrals.

### Eligibility criteria {10}

Inclusion and exclusion criteria for study participants are detailed in Table 1.

**Table 1:** Inclusion and exclusion criteria for caregivers

Inclusion	Exclusion
Age ≥ 18 years	Caregiver actively receiving psychiatric/psychologist care identified through self-report prior to consent
Informal caregiver of a person living with MND at home who is in the later stages of the disease, as evidenced by the need for consideration or receiving any of the following (i.e. entering King’s clinical staging Stage 4A: nutritional support; or Stage 4B: respiratory support): (a) Assisted ventilation (b) Cough assist (c) Gastrostomy and enteral feeding	
Able to speak/read English	
Consents to participation	

Peer supporters are required to have lived experience of caring for a person living with MND, able to speak/read English, and provide informed consent

### Who will take informed consent? {26a}

Participant Identification Centres will identify potentially eligible participants and provide the participant information sheet (PIS), discuss the study, and obtain consent-to-contact from eligible participants who wish to be contacted by a member of the study team. Informed consent will be obtained either over the telephone by a member of the central research team or via a Qualtrics digital consent form sent to participants via email. The PIS and consent materials are available from the corresponding author on request.

### Additional consent provisions for collection and use of participant data and biological specimens {26b}

Not applicable. Participant data will not be used in ancillary studies and no biological specimens will be required in the study.

### Interventions

#### Explanation for the choice of comparators {6b}

Participants randomised to the control arm will be provided with a standard list of the free support resources available from the MND Association website. They will be made aware of the MND Association Visitors programme and MND Association’s publicly available online educational resources.

#### Intervention description {11a}

Participants randomised to the intervention arm will be provided access to a 12-week virtual peer-to-peer support programme comprising (1) audio, video, or text private and secure one-to-one messaging with a dedicated peer supporter; (2) moderated synchronous weekly discussion forum for peer supporters and informal caregiver participants on specific topics (e.g. caregiver self-care, the emotional impact of caregiving); (3) asynchronous group chats in which participants can continue discussions and post questions; and (4) access to informational resources.

#### Intervention delivery

The 12-week virtual peer support programme will be hosted via the secure digital e-platform aTouchAway (Aetonix, Canada). Each participant will be assigned a peer supporter by the research team. Participants will be advised to engage with their peer supporter at a minimum of once a week and utilise all elements of the programme including text messaging, audio/video calling, and group chat features of the aTouchAway app. Participants will also be expected to join a minimum of seven of the 12 weekly synchronous discussion forums.

#### Intervention training

Peer supporters with lived experience of caring for a person with MND will receive training from the research team and will receive a virtual peer-to-peer support programme manual. Training will include four 90-min virtual training sessions covering topics such as responsibilities as a peer supporter, boundary setting, managing risk, empathetic communication, and peer support at end of life, as well as use of the aTouchAway app. A check in/debrief session with the study team will be offered to peer supporters every 4 weeks. Intervention participants will receive a one-to-one introduction to the virtual peer support programme and a resource manual on the peer support intervention.

#### Criteria for discontinuing or modifying allocated interventions {11b}

The study does not involve patients as participants and the intervention is considered low risk. Therefore, we do not anticipate the need to discontinue or modify the intervention itself. We do not anticipate serious adverse events resulting from the intervention; however, it is possible that participants may experience psychological distress due to their caregiving situation. In such cases, we will advise the study participant to make a GP appointment. Given the rapidly progressive nature of MND, it is feasible that a caregiver may experience death of the person for whom they provide care. In this event, we will ask the participant if they still wish to continue receiving peer support. Request to make a GP appointment and death of a care recipient will be documented as an adverse event. Peer supporters may experience difficulties or conflict with a study participant and may require reassignment. In additional, unanticipated life events may result in a peer supporter to request to withdraw from the role. We will document these events as adverse events. All peer supporters will be given the opportunity to participate in the semi-structured qualitative interviews exploring their experience as a peer supporter.

#### Strategies to improve adherence to interventions {11c}

We will instruct peer supporters to set expectations and boundaries in terms of frequency and timing of contact in accordance with their personal preferences. We will also instruct peer supporters to contact their allocated study participant a minimum of once a week and let the research team know if no contact is received. Participants are provided the same instructions. Peer supporters will have access to a senior clinical member of the research team throughout the programme with whom they can share any concerns about themselves or their assigned participant. The research team will provide a peer supporter debriefing session every 4 weeks to give them an opportunity to discuss any issues in relation to their peer supporter role within the trial and to discuss solutions.

#### Relevant concomitant care permitted or prohibited during the trial {11d}

Participation in other studies is not an exclusion but will be assessed by the research team for appropriateness, i.e. participation in a research trial of another intervention designed to provide psychological support. Participants will not be prohibited from accessing other support resources such as the MND Association Visitor programme or family caregiver drop-in sessions hosted by other organisations. We will only enrol one informal caregiver participant for each individual with MND.

#### Provisions for post-trial care {30}

Participants will continue to receive usual care after their involvement in the trial. Those participants requiring ongoing support will be signposted to the MND Association Visitors programme and the open educational resources available publicly on their website. Although participants randomised to the peer support programme will have the aTouchAway app disconnected upon programme completion, we will not prohibit informal arrangements to continue a peer support relationship if the peer supporter is in agreement.

#### Outcomes {12}

The primary outcome is as follows: the study primary outcome is total HADS score at 12 weeks. The HADS is assessed at baseline and 6 and 12 weeks to compare the two treatment groups. The HADS consists of a 14-item self-report scale with seven items on the anxiety subscale (HADS-A) and seven items on the depression subscale (HADS-D) [18]. Each item is scored on a 4-point (0–3) response category scale with total scores ranging from 0 [best] to 21 [worst] for each subscale, with cutoff points of > 7 and > 10 indicating possible or probable anxiety and depression. Two items from the HADS-A and four items from the HADS-D are reversed scored. The HADS was selected as the primary outcome as it has been extensively validated and used previously in numerous caregiver populations [19], including caregivers of people with MND [20]. Importantly, it has previously been used in studies that evaluated the effect of internet-based interventions designed to mitigate negative mental health outcomes associated with caregiving [21]. The HADS is also the primary outcome of a randomised controlled trial on the effectiveness of a blended psychosocial support programme for informal caregivers of individuals with Amyotrophic Lateral Sclerosis [22]. The HADS has good acceptability, reliability, (Cronbach’s alpha from 0.72 to 0.93), validity, and discrimination [23].

The secondary outcomes are as follows: Pearlin’s stress process model of informal caregiving [24] informed selection of secondary outcome measures. This model considers various factors which may interact and determine how an individual reacts to the role of caregiving. These are as follows: (1) contextual factors (e.g. sociodemographic characteristics) or variables related to caregiving (e.g. duration of caregiving role); (2) primary stress factors directly related to the health of the care recipient and the degree of the care needed; (3) secondary stress factors beyond the caregiving role such as restriction of social life, difficulties at work, or financial strain; and (4) mediating and moderating factors that can determine how well an individual copes with their caregiver role. Stress factors may be objective (e.g. cognitive impairment) or subjective (e.g. perception of overload). These factors, together with coping strategies, personal mastery, social support, beliefs, and values influence health outcomes such as well-being, depression, anxiety, burden, and may account for variability in the health consequences the caregiver experience.

We will use the Zarit Burden Interview (ZBI) [25] to assess subjective burden or stress associated with caregiving. The ZBI consists of 22 items with five ordered frequency-related response categories scored 0 (never) to 4 (nearly always). All 22 items are used to calculate a total score that ranges between 0 and 88. A score of ≥ 24 reflects high burden; a score of < 24 indicates low burden [26, 27]. We will use the 10-item Positive Affect Scale of the Positive and Negative Affect Schedule [PANAS] [28] to measure positive affect. Scores range from 10 to 50; higher scores indicate better psychological well-being. The Pearlin Mastery Scale [29] is a 7-item scale with scores from 7 to 28. We will use this scale to assess caregiver sense of control over life. Individuals with a high sense of mastery believe in their power to influence the environment and bring about desired outcomes; those with low mastery feel less able to control events and circumstances of their lives [30]. The Personal Gain Scale [24] is a 4-item scale with scores range from 4 to 16. We will this scale to determine discovery of inner strengths through providing care. The Brief COPE [31] scale is a 28-item scale grouped into three overarching coping styles: problem focused, emotion focused, and avoidant. This scale will be used to assess frequency of various coping strategies. The Caregiver Assistance Scale (CAS) [32, 33] will be used to determine any changes in the level of assistance with daily activities and medical care provided by the informal caregiver. Scores on the CAS range from 0 to 102, with higher scores indicating more caregiving assistance. The Caregiving Impact Scale (CIS) [33] will be used to determine the impact of provision of care on the caregivers’ ability to maintain participation in valued activities. Scores on the CIS range from 0 to 84. Higher scores indicating that providing care interferes with caregivers’ abilities to maintain participation in valued activities.

Additionally, participants randomised to the intervention arm will be asked to rate the usability of the virtual peer-to-peer support programme on a 9-point Likert scale ranging from difficult to easy. Participants will be asked to perform this rating on completion of the 12-week programme. Intervention fidelity will be assessed by (1) determining the extent of participant contribution to the three facets of the peer support programme: synchronous discussion forum, private chat, and asynchronous group chat; (2) evaluating communication activities, i.e. number (text), and number and duration of audio and video calls; and by (3) determining attrition rate and reasons for study discontinuation.

#### Data collection

We will collect demographic and caregiving history from both trial participants and peer supporters. Data will include age, gender, relationship to the person with MND they care for (or cared for in the case of peer supporters), educational level, employment status, previous experience with caregiving, and duration of their caregiving relationship. For trial participants, we will also collect data on the number of informal (unpaid) caregivers for the person with MND they provide care for and the amount (hours) of formal (paid) caregiving received from health and/or social care workers. We will collect the following measures at baseline and at 12 weeks (programme end): HADS, PANAS, ZBI, Pearlin Mastery Scale, Personal Gain Scale, Brief COPE, CAS, and CIS (only at baseline). We will also collect the HADS and the ZBI at 6 weeks (programme midpoint).

Demographics data and participant-reported outcome measures will be collected using a secure web-based survey platform (Qualtrics). Participants will be sent Qualtrics autogenerated secure questionnaire links at the study time points. Participants not comfortable with e-questionnaires will be provided the opportunity to answer the questionnaires over the telephone with the blinded research officer. If requested, we will also post copies of questionnaires. All data entered into Qualtrics will be exported to the study database hosted on the electronic data capture (EDC) system Castor v.15.81 [34].

We will collect the following endpoints at 12 weeks (programme end) from intervention arm participants only: (1) perceived usability of the peer-to-peer support programme using a 1-item 9-point Likert Scale using a Qualtrics autogenerated questionnaire link emailed to intervention participants; (2) acceptability of the peer-to-peer support programme (in a subset of intervention participants and peer supporters) via semi-structured qualitative interviews (digitally recorded and professionally transcribed) using topic guides informed by the Theoretical Framework of Acceptability (TFA) [35, 36]; and (3) usage metrics for both intervention participants and peer supporters will be downloaded from the aTouchAway platform. Usage metrics will comprise the following: (i) the frequency and type (text, audio, video) of peer-to-peer contact each week and overall for the 12-week programme; for audio and video contacts, we will also capture the duration of contact; (ii) the frequency of participation in a synchronous or asynchronous discussion forum; and (iii) discontinuation of the programme before 12 weeks and the week discontinued.

We will offer the opportunity to participate in a one-on-one telephone semi-structured interview to intervention participants and peer supporters, until we reach data saturation. Maximum variation sampling will be used to ensure a minimum of two participants representing caregiver variables including age (≤ 65; > 65), sex (male; female), and relationship to care-recipient (spouse, child, parent, other). We will offer the opportunity to participate in an interview at the end of a 12-week intervention period to study participants and peer supporters.

### Participant timeline {13}

The participant timeline is shown in Table 2.

**Table 2:**
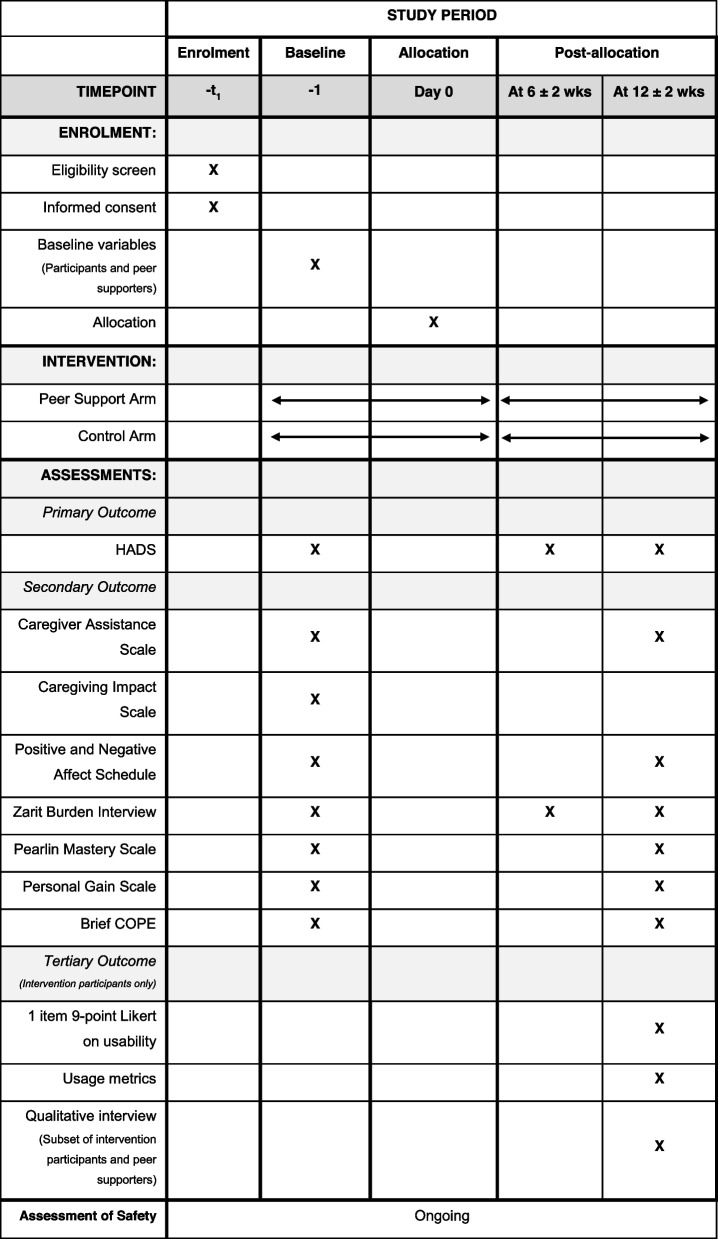
Schedule of enrolment, intervention, and assessments

#### Sample size {14}

To determine sample size estimates, we used the sample size calculation method used in the previously published De Wit trial [22]. The De Wit trial sample size is based on HADS data from a longitudinal study of MND informal caregivers [4]. To test for a medium size effect (*d* = 0.5) at 5% level of significance (two-sided) with 80% power would require 64 participants in each arm (128 in total) [22]. To find an adjusted sample size “*N*” from a calculated sample size “*n*” and an estimated attrition rate “*x*”, we used the following formula: *N* = *n*/(1-*x*). We adjusted the sample size for 20% attrition to get a total sample size of 128/(1–0.2) = 160 (80 in each arm). We determined the sample size for the semi-structure qualitative interviews using recommendations for deciding saturation in theory-based interview studies [37]. We will recruit a minimum sample of 20–30 participants and 15–20 peer supporters. We will adjust the sample size using a stopping criterion of three consecutive interviews with no additional material to terminate data collection.

#### Recruitment {15}

We will use a multi-modal recruitment strategy to recruit informal caregiver participants including MND clinics, home ventilation clinics, and hospices across England, Scotland, and Wales as well as via the UK MND Association, social media, and snowballing referrals. The research team will recruit participants directly from the MND clinics of the main study site. Participant Identification Centre sites will provide study recruitment materials (recruitment flyer and the participant information sheet) to potential participants during their attendance accompanying their relative with MND to a virtual or in-person clinic appointment. Consent-to-contact will be collected from family caregivers expressing interest in participation. Interested informal caregivers will then be contacted by the central research team to explain the study, to obtain informed consent, and to collect baseline data. The MND Association and Marie Curie Charity will advertise on their websites, newsletters, and discussion forums. We will also post recruitment notices and study updates on our dedicated Twitter account. We will post trial information on other relevant forums including Health Watch-UK, healthtalk.org, and the King’s College London research volunteer newsletter. We will encourage snowballing recruitment methods. Peer supporters will be primarily recruited from the MND Association Visitors programme and other MND Association awareness campaigns.

### Assignment of interventions: allocation

#### Sequence generation {16a}

Participants will be randomised on a one-to-one basis to the intervention arm or control arm via Castor Electronic Data Capture (EDC) using a computer-generated allocation sequence based on variable block sizes (4, 6, 8) with stratification per caregiver gender (female vs other).

#### Concealment mechanism {16b}

The unblinded research officer will perform randomisation in Castor EDC and subsequently notify the participant of their group allocation. Allocation will be concealed using this computer-generated mechanism. The unblinded research officer will have no role in outcome assessment.

#### Implementation {16c}

**Fig. 1:**
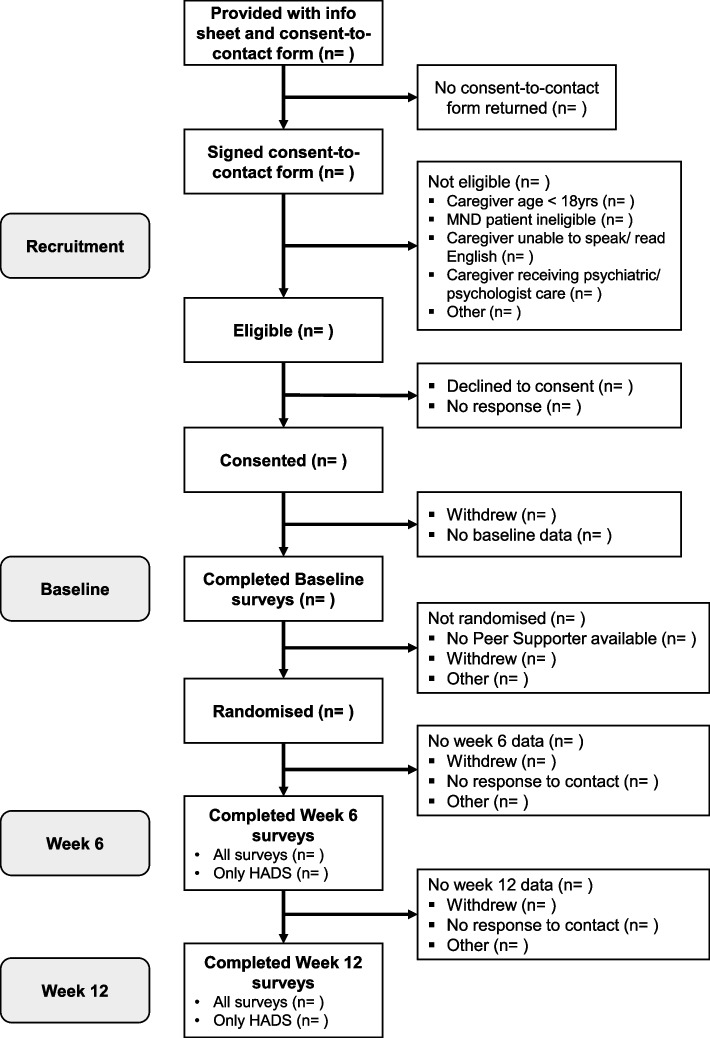
Flow diagram of implementation sequence

The unblinded research officer will enter the stratification data (participant’s gender obtained from baseline demographics) into the study database hosted on the Castor EDC. The randomisation module will be used to randomise the participant. If allocated to the intervention arm, the unblinded research officer will provide the participant access to the peer support programme via the aTouchAway app and assign a peer supporter. The unblinded research officer will be the point of contact for mentors and participants in the intervention arm. If allocated to the control arm, the unblinded research officer will signpost the participant to the MND Association’s publicly available informational resources and volunteer programme. The implementation sequence is shown in Fig. 1.

### Assignment of interventions: blinding

#### Who will be blinded {17a}

The research officer responsible for outcome assessment and the study statisticians will be blinded to group allocation. As we are unable to blind participants and peer supporters due to the nature of the intervention, we will instruct participants not to disclose their allocation status when providing outcome data over the telephone to the blinded research officer.

#### Procedure for unblinding if needed {17b}

Not applicable. Due to the low-risk nature of the intervention there will be no circumstances during the trial under which unblinding will be required.

### Data collection and management

#### Plans for assessment and collection of outcomes {18a}

Data will be collected at specified time points (see Table 2) using pre-designed case report forms over the phone, via the Qualtrics platform or postal mail depending on the participant’s stated preference. For participants who require a break during data collection over the phone, a second call will be scheduled to complete collection of data collection measures.

#### Plans to promote participant retention and complete follow-up {18b}

The main strategy to promote retention will include communication from the study team at discussion forums with participants and peer supporters. We are also offering a multi-modal strategy to provide outcome data including via emailed Qualtrics link, telephone, or postal options to improve response to outcome questionnaires. We included measurement of the HADS and ZBI at 6 weeks as another form of contact.

#### Data management {19}

We will use Castor EDC for valid data collection and secure data storage. The web-based study database will be located on a UK server, password protected and only accessible to the unblinded research officer.

#### Confidentiality {27}

In accordance with the General Data Protection Regulation (GDPR) and Data Protection Act 2018, all stored electronic data will be deidentified and stored on the password protected King’s College London server. A unique participant study number will be assigned to each participant and used on all study documents and any electronic database(s) to minimise processing of personal data. The UK Aetonix AWS cloud will store anonymised data on participant interaction with the peer-to-peer support programme (i.e. the number, duration, and type of interactions). This will be downloaded for each participant on study completion and stored in the Castor database.

Qualitative data from semi-structured interviews will be digitally recorded on an encrypted recorder and the files will be sent securely to a professional transcribing company. Electronic files of transcription will be password protected and stored on the password protected King’s College London server. Personal data used for sending questionnaires and setting up interviews will be securely destroyed on completion of study participation. Research data will be stored for 10 years. Data will be handled and stored in accordance with the 2018 Data Protection Act.

#### Plans for collection, laboratory evaluation, and storage of biological specimens for genetic or molecular analysis in this trial/future use {33}

This is not applicable as no biological specimens will be collected.

### Statistical methods

#### Statistical methods for primary and secondary outcomes {20a}

We will conduct analyses on an intention to treat (ITT) basis according to a pre-specified statistical analysis plan (SAP) authored by the trial statistician and approved by the trial steering committee prior to database lock. The ITT population will be defined as all participants randomised with at least one post-baseline outcome assessment of the HADS. The per protocol population (PPP) will be defined as all patients meeting the ITT that adequately followed the protocol. Participants deemed to have breached the protocol will be recorded as protocol violators and removed from the PPP. To test the effect of the peer-support intervention on the primary outcome (caregiver psychological distress as measured by the HADS total score), we will use a mixed effects linear model adjusting for the fixed effects of HADS at baseline, group allocation (intervention/control), baseline caregiving assistance scale score, baseline Caregiving Impact Scale scores, number of informal caregivers, age, gender (female/other), and if the patient died during the study before the final follow-up. Participants will be fitted with random effect across both week 6 and 12 HADS measurement. The analysis will be presented as the adjusted mean difference between intervention and control arms with a 95% confidence interval and *p*-value at 12 weeks.

The ZBI captured post-randomisation at 6 weeks and at 12 weeks will be analysed using mixed effects linear model adjusting for fixed effects as per the primary outcome analysis. Secondary continuous outcomes captured post-randomisation at 12 weeks (Positive Affect Scale, Pearlin Mastery Scale, Personal Gain Scale, Brief COPE total score) will be analysed using linear models with random effect for participant but without the same fixed effects as in the primary analysis model. Binary outcomes such as proportion of participants scoring > 10 in the HADS-A and HADS-D will be assessed using logistic regression models in a similar manner as the primary analysis and presented as adjusted odds ratios with 95% confidence intervals. All tests will be two sided. A *P* value of < 0.05 will be considered statistically significant for the primary outcome. We will use Stata (V15 or higher) and R for analyses and graphical representation.

We will employ a directed content analysis approach for the qualitative analysis, as outlined by Bengtsson [38]. Summative content analysis will be performed to contextualize patterns in the data informed by the Theoretical Framework of Acceptability (TFA) [35]. Data will be coded using the seven TFA domains: affective attitude, burden, ethicality, intervention coherence, opportunity costs, perceived effectiveness, and self-efficacy.

#### Interim analyses {21b}

This is not applicable. Interim analyses will not be performed given this is a low-risk intervention study.

#### Methods for additional analyses (e.g. subgroup analyses) {20b}

We will perform a sensitivity analysis excluding data from caregivers who continue participation in the study despite death of the individual with MND for whom they provide care.

#### Methods in analysis to handle protocol non-adherence and any statistical methods to handle missing data {20c}

We will assess variables for missing data. If post randomisation variables such as intervention compliance (minimum of a once a week contact with peer supporters plus participation in a minimum of 7/12 synchronous discussion forums) are seen to predict missingness of the primary outcome, then we will consider multiple imputation using chained equations (MICE). We will assess model residuals for normality. We will perform a bootstrap analysis if the residuals (observed minus predicted value) deviate noticeably from normality. In the event of a very low intervention compliance, a complier average causal effect (CACE) analysis for the primary HADS outcome will be considered to investigate the effect on individuals who complied with the intervention. Additional sensitivity analyses addressing potential intercurrent events and non-compliance will be outlined in the statistical analysis plan.

#### Plans to give access to the full protocol, participant level-data, and statistical code {31c}

The final dataset will be available from the research team upon reasonable request. We will use the CONSORT-SPI 2018 Extension reporting guidelines [39] to ensure comprehensiveness and transparency of study findings. We will use a CONSORT-SPI flow diagram to describe participant flow through the study including the number of participants approached for enrolment, the decline rate, and the assessments to confirm eligibility and outcomes between groups. To avoid selective reporting, all outcomes will be reported as outlined in this study protocol.

### Oversight and monitoring

#### Composition of the coordinating centre and trial steering committee {5d}

The trial management group (TMG) will comprise of the chief investigator (CI), research officers, and the trial statistician team. The TMG (CI and research officers) will meet every week with the statisticians joining monthly to ensure the study is progressing according to protocol, planned timelines, and budget. The TMG will prepare a report for the trial steering committee (TSC) who will provide expert oversight and advice on all aspects of the trial.

The composition of the TSC will be based on the Medical Research Council recommendation for TSC membership and will include (1) the chief investigator (non-independent of the trial), (2) an independent chair (with clinical expertise in the field of MND and research expertise in the conduct of clinical trials), (3) an independent statistician, (4) two independent representatives one of whom is a former caregiver, and (5) a non-independent representative of the MND Association. The TSC will meet at least every 12 months to review recruitment, dropout rates, and safety data as provided by the study statistician.

The role of the TSC will include, but not limited to, the following: oversee participant rights, safety, and well-being, review regular reports of the study, assess data quality including completeness (and by so doing encourage collection of high-quality data), comment on the project analysis plan, comment on the publication policy, oversee the timely reporting of study results, monitor compliance with the protocol and any amendments, monitor compliance with previous TSC recommendations, monitor recruitment, and encourage the TMG to develop strategies to deal with any recruitment problems. Meetings will be held by videoconference unless a face-to-face meeting is deemed necessary in agreement with CI and TSC chair.

#### Composition of the data monitoring committee, its role and reporting structure {21a}

Due to the low-risk nature of the intervention we will not convene an independent Data Safety Monitoring Committee.

#### Adverse event reporting and harms {22}

As the study does not involve patients and involves a low-risk intervention (i.e. peer support programme) with informal caregivers, we do not anticipate serious adverse events as a consequence of participation in the study.

We will consider any occasion when a participant is advised by the study team or peer supporter to contact their GP as an adverse event. The research team will advise a participant to contact their GP or other relevant supports if increased psychological distress is detected with a depression and/or anxiety (cut-off of score > 10) in the HADS measured at 6 and 12 weeks or if a peer supporter raises concern.

In the event of concern about a participant, the research team may recommend the following options: (1) ask the participant if a family member or friend can be contacted for immediate support; (2) recommend the participant contact their GP for further support; and (3) recommend the participant accesses other avenues of support such as that offered by the MND Association Visitors programme or Marie Curie. Peer supporters will be advised to contact the unblinded research officer if they have concerns warranting advice to a study participant to contact a GP.

An adverse event will be documented on any occasion when a peer supporter expresses difficulty or conflict with their allocated participant that requires intervention from the research team or when a peer supporter requests to withdraw from the peer supporter role during the 12-week intervention period. Any expression of conflict or difficulty will be adjudicated in terms of required action and may include the following: (1) no need for action, informal debrief sufficient, (2) need for the unblinded research officer to reiterate to participant the expectations regarding engagement with their peer supporter, (3) reassignment to another peer supporter, and (4) discontinuation from the trial. We will also consider death of the person a participant is providing care for as an adverse event.

In the event of an SAE occurrence, it will be reported immediately and always within 24 h upon knowledge of the event to the sponsoring R&D department. We will submit SAE reports to the Main REC within 15 days of the chief investigator becoming aware of the event, using the National Research Ethics Service (NRES) template. All other AEs will be reported to the sponsor in the Annual Progress Report and included in the TSC committee report for review.

#### Frequency and plans for auditing trial conduct {23}

Not applicable. Audits will not be part of this trial. Routines meetings will be held by the TSC no longer than annually to review trial conduct and progress. The TMG will meet monthly to ensure that the study is being conducted in accordance with the study protocol. The REC will not review conduct during the trial period. We will provide an annual progress report to the REC that details recruitment, adverse events, amendments and protocol deviations.

#### Plans for communicating important protocol amendments to relevant parties (e.g. trial participants, ethical committees) {25}

We will obtain sponsor approval for all substantial and non-substantial amendments to the original approved documents. Substantial amendments will be submitted to the research ethics committee (REC) for written approval. Protocol amendments will be updated on the Clinical Trial register. We will not communicate amendments with trial participants. We will submit an Annual Progress report to the REC, HRA (where required), study sponsor, and funder. We will provide an End of Study notification and final report to the same parties.

#### Dissemination plans {31a}

We will report the primary trial result and process evaluation of the RCT as manuscripts submitted to peer reviewed journals, with presentation at relevant academic conferences. We will email all study participants a lay result summary. We will send an executive summary to the MND Association and Marie Curie.

## Discussion

In this trial, we will investigate the effect of a peer support programme delivered through the e-platform aTouchAway™ on informal caregiver psychological health and caregiver burden. The rapid and progressive nature of the MND cause psychological distress to informal caregivers of individuals and can intensify informal caregiver feelings of helplessness and need for psychosocial support [20]. Digital interventions can provide informal caregivers with remote access to a psychosocial support network and may contribute to a more positive caregiving experience. We hypothesise our 12-week virtual peer support programme will improve psychological health and alleviate caregiver burden.

To tailor the peer support programme in accordance with MND caregiver needs, we have collaborated with the MND Association, caregiver advisory groups and consulted with several informal caregivers over the past two years. Anticipated challenges include time constraints for caregivers to participate in peer support and provide study data as well as low digital literacy. We will facilitate data collection through multi-modal and flexible methods. Digital inclusion will be supported though loaning of 4G enabled Android tablets to those without devices if required. In summary, this trial will provide important data on a digital peer support programme specifically designed as a tool to improve psychological health and support informal caregivers of individuals living with MND.

### Trial status

Patient recruitment began in June 7, 2022. The planned recruitment closure date is August 2, 2024. The current protocol version (version 1.2) is dated March 11, 2022.

## Data Availability

The final dataset will be available on request.

## Abbreviations

ALS: Amyotrophic lateral sclerosis
CAS: Caregiver Assistance Scale
CI: Chief investigator
CIS: Caregiving Impact Scale
EDC: Electronic data capturing
HADS: Hospital Anxiety and Depression Scale
HRA: Health Research Authority
Main REC: Main Research Ethics Committee
MND: Motor neurone disease
MNDA: MND Association
NRES: National Research Ethics Service
PANAS: The Positive and Negative Affect Schedule
Participant: An individual who takes part in a clinical trial
PI: Principal investigator
PIC: Participant Identification Centre
RCT: Randomised controlled trial
REC: Research ethics committee
SAE: Serious adverse event
TFA: Theoretical Framework of Acceptability
TMG: Trial management group
TSC: Trial steering committee
ZBI: Zarit Burden Interview
